# Global diabatic potential energy surfaces and quantum dynamical studies for the Li(2p) + H_2_(X^1^Σ^+^_g_) → LiH(X^1^Σ^+^) + H reaction

**DOI:** 10.1038/srep25083

**Published:** 2016-04-29

**Authors:** Di He, Jiuchuang Yuan, Huixing Li, Maodu Chen

**Affiliations:** 1 Key Laboratory of Materials Modification by Laser, Electron, and Ion Beams (Ministry of Education), School of Physics and Optoelectronic Technology, Dalian University of Technology, Dalian 116024, PR China

## Abstract

The global diabatic potential energy surfaces which are correlated with the ground state 1A′ and the excited state 2A′ of the Li(2p) + H_2_ reaction are presented in this study. The multi-reference configuration interaction method and large basis sets (aug-cc-pVQZ for H atom and cc-pwCVQZ for Li atom) were employed in the *ab initio* single-point energy calculations. The diabatic potential energies were generated by the diabatization scheme based on transition dipole moment operators. The neural network method was utilized to fit the matrix elements of the diabatic energy surfaces, and the root mean square errors were extremely small (3.69 meV for 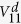, 5.34 meV for 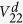 and 5.06 meV for 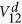). The topographical features of the diabatic potential energy surfaces were characterized and the surfaces were found to be sufficiently smooth for the dynamical calculation. The crossing seam of the conical intersections between the 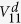 and 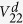 surfaces were pinpointed. Based on this new analytical diabatic potential energy surfaces, time-dependent wave packet calculation were conducted to investigate the mechanism of the title reaction. At low collision energies, the product LiH molecule tends to forward scattering, while at high collision energies, the forward and backward scatterings exist simultaneously.

Chemical reactions involving Li atom and H_2_ molecule play an important role in early cosmic evolution^1^,^2^,^3^,^4^,^5^,^6^,^7^,^8^,^9^,^10^. Moreover, LiH molecule makes a considerable contribution to the cosmic background radiation^7^,^8^,^9^. The elementary reactions Li(2s, 2p) + H_2_(X^1^Σ^+^_g_) ↔ H + LiH(X^1^Σ^+^) have great influence on the formation and depletion of LiH^7^,^8^. Therefore, the research concerning the LiH_2_ system plays a vital role in understanding the early evolution of the universe.

Over the past decades, the ground state of the LiH_2_ system has received extensive attention in theoretical studies^9^,^10^,^11^,^12^,^13^. The first three-dimension adiabatic potential energy surface (PES) for the ground state of the LiH_2_ molecule was constructed by Dunne *et al.*^11^. This PES shows an unphysical well at the Li(2s) + H_2_(X^1^Σ^+^_g_) asymptotic valley. In 2009, two PESs of the ground states of LiH_2_ system were reported by Prudente *et al.* and Wernli *et al.*^12^,^13^ Recently, Chen and co-workers presented a high-precision PES for the ground state of the LiH_2_ system^14^. All the four PESs were employed to study the reaction dynamics of the LiH(X^1^Σ^+^) + H → Li(2s) + H_2_(X^1^Σ^+^_g_) reaction using the quasi-classical trajectory (QCT) and time-dependent wave packet (TDWP) method^12^,^15^,^16^,^17^.

Although the LiH(X^1^Σ^+^) + H → Li(2s) + H_2_(X^1^Σ^+^_g_) reaction has been widely studied in theory, less attention has been paid to experimental studies. In contrast, a large number of experimental studies have been carried out on the Li(2p) + H_2_(X^1^Σ^+^_g_) → LiH(X^1^Σ^+^) + H reaction^18^,^19^,^20^,^21^. Mysers *et al.*^18^ measured the reaction cross section (σ = 0.10 ± 0.03 Å^2^) of the Li(2p) + H_2_(X^1^Σ^+^_g_) reaction at a temperature of *T* = 788 K. In 2001, Lin and co-workers^19^ measured the rotational population distribution of LiH(*v* = 0) for this reaction by using a pump–probe technique, and observed that the reactive collisions are dominated by insertion mechanism. Bililign *et al.*^20^ observed that the Li(2p) + H_2_ reaction occurs preferentially within bent geometry (near *C*_*2v*_) by using the far-wing scattering spectroscopy. Chen *et al.*^21^ studied the effect of vibrational excitation of H_2_ on the rotational state distributions of LiH(*v* = 0) for the Li(2p) + H_2_(*v* = 1) → LiH(X^1^Σ^+^) + H reaction using a pump-probe technique.

Although, many experimental studies have focused on the Li(2p) + H_2_(X^1^Σ^+^_g_) → LiH(X^1^Σ^+^) + H reaction, few theoretical studies of this reaction have been reported^22^,^23^. The reactants Li(2p) + H_2_(X^1^Σ^+^_g_) and the products LiH(X^1^Σ^+^) + H correlate with two different adiabatic states (2^2^*A*′, 1^2^*A*′). Thus, the Born-Oppenheimer (BO) approximation is not valid in the vicinity of the conical intersection^24^. To appropriately deal with the breakdown of the BO approximation, a proper diabatization scheme should be chosen to adequately treat the coupling between the two states. In 1999, Lee *et al.*^22^ investigated the Li(2p) + H_2_ collisions. The PES used in their research is limited to three different point group symmetries: *C*_∞*v*_, *C*_2*v*_ and *C*_*s*_ where the molecular axis of the H_2_ molecule makes an angle of π/4 with respect to the velocity vector of the lithium atom. Since this PES is not global, it cannot accurately describe all the features of the intermolecular interaction potential of the Li(2p) + H_2_ reaction. In 2011, two adiabatic global PESs of LiH_2_ (1^2^*A*′, 2^2^*A*′) were constructed by Hsiao *et al.*^23^, and the QCT calculations were carried out based on their PESs. In their QCT calculations, the trajectories initially evolve on the excited state surface 2^2^*A*′ and hop to the ground state 1^2^*A*′ at the exit channel. The transition probability between the two states (1^2^*A*′, 2^2^*A*′) was assumed to be unity, which is an approximate method for treating the diabatic process of the reaction. A valid method for describe the effect of the diabatic process for the molecular reaction accurately, is to construct a set of diabatic PESs for the coupled electronic states. However, for the title reaction, there are no such a set of diabatic PESs that can be employed for dynamical calculations. To meet these requirements above mentioned, a set of global diabatic PESs was constructed for the lowest two ^2^*A*′ states of LiH_2_ system using the neural network (NN) method^25^. Based on the diabatic PESs, the reaction dynamics of the title reaction were investigated using the TDWP method.

## Results

### PESs topographical attributes

**Figure 1:**
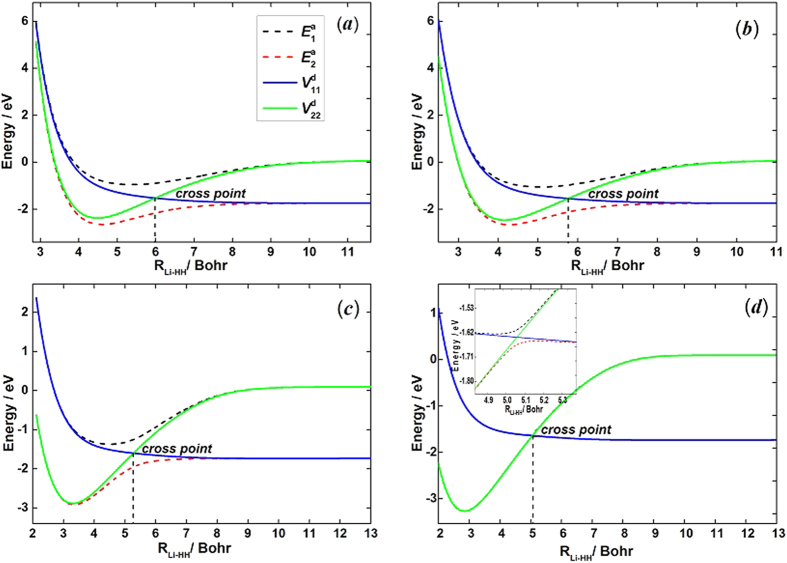
Adiabatic and diabatic potential energies at (**a**) *θ* = 3.0°, (**b**) *θ* = 30.0°, (**c**) *θ* = 60.0°, (**d**) *θ* = 90.0° for fixed *r*_*HH*_ = 2.9 *a*_0_ vs *R*_Li-HH_. The solid lines represent the diabatic potential energies and the dashed lines represent the adiabatic potential energies.

**Figure 2:**
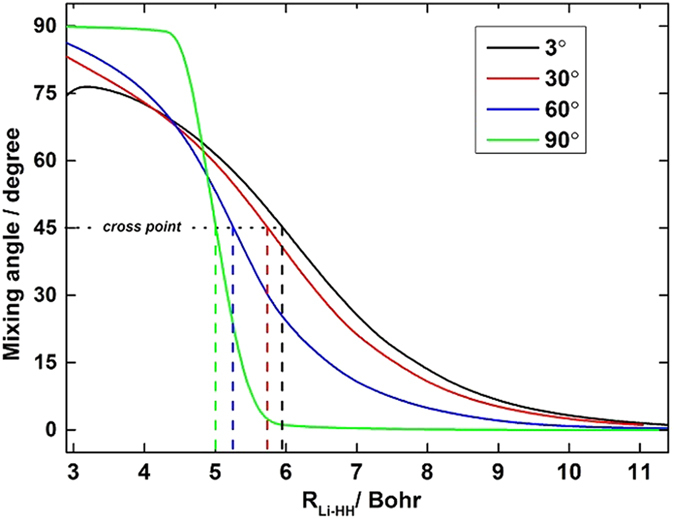
Mixing angles as function of *R*_Li-HH_ for fixed *r*_*HH*_ = 2.9 *a*_0_ and (**a**) *θ* = 3.0°, (**b**) *θ* = 30.0°, (**c**) *θ* = 60.0°, (**d**) *θ* = 90.0°.

**Figure 3:**
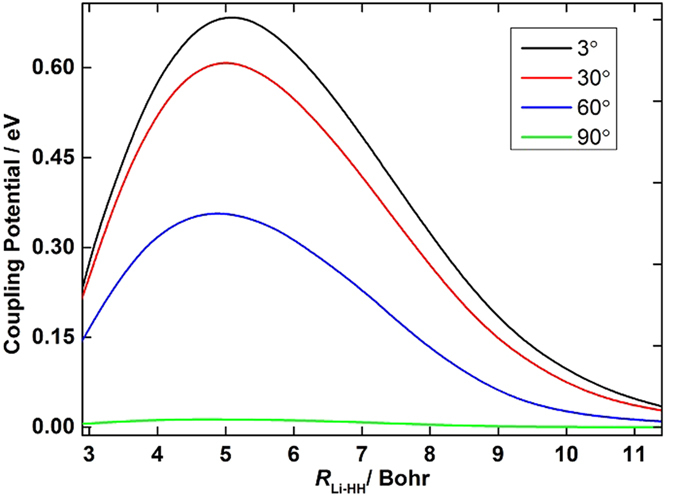
Coupling potentials as function of *R*_Li-HH_ for fixed *r*_*HH*_and (**a**) *θ* = 3.0°, (**b**) *θ* = 30.0°, (**c**) *θ* = 60.0°, (**d**) *θ* = 90.0°.

**Figure 4:**
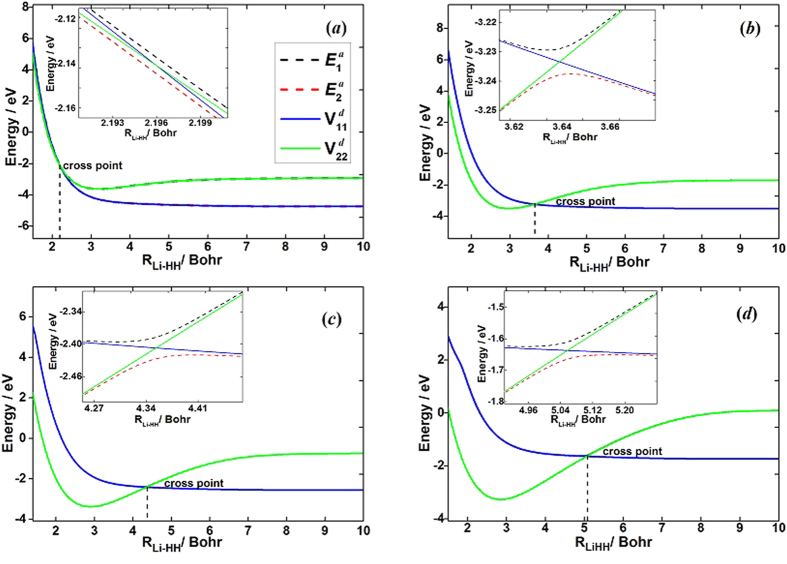
Adiabatic and diabatic potentials at (**a**) *r*_*HH*_ = 1.4 *a*_0_, (**b**) *r*_*HH*_ = 2.1 *a*_0_, (**c**) *r*_*HH*_ = 2.5 *a*_0_, (**d**) *r*_*HH*_ = 2.9 *a*_0_ for fixed *θ* = 90.0° vs *R*_Li-HH_. The solid lines represent the diabatic energies and the dashed lines represent the adiabatic energies.

**Figure 5:**
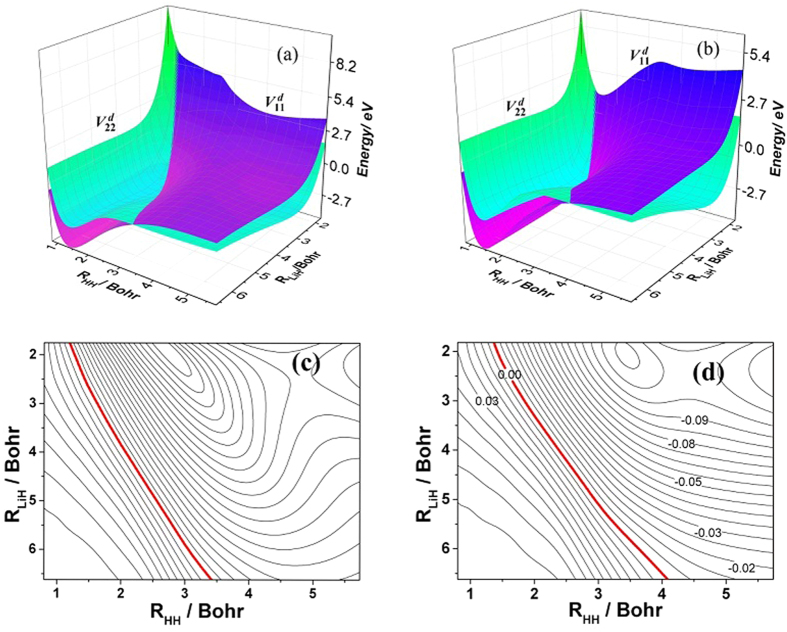
Three dimension PESs of the diabatic states 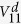 and 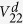 for Li-H-H with a bond angle of (**a**) 60.0° and (**b**) 90.0°. The corresponding contour plot of their differences (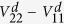) are showed in (**c**,**d**), respectively. The red lines represent the position of the diabatic crossing.

**Figure 6:**
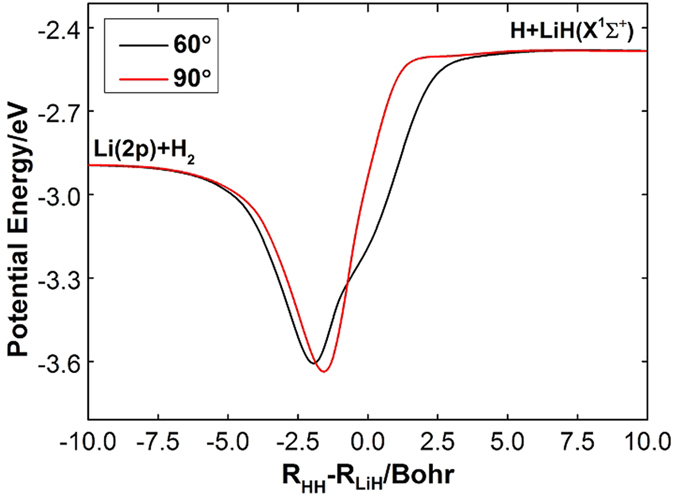
Minimum energy paths of diabatic PES 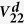 for Li-H-H angle fixed at 60.0° and 90.0°.

The accuracy of the *ab initio* computations can be assessed by comparing the calculated spectroscopic constants with the experimental values. The spectroscopic constants of H_2_ and LiH obtained from the diabatic PESs and the corresponding experimental data are listed in Table 1. The H_2_ and LiH spectroscopic constants were calculated in the supermolecule approach with the other atom (Li and H, respectively) at a distance of 30 *a*_0_. Obviously, all of the theoretical results have a good agreement with the corresponding experimental results^26^,^27^. The calculated equilibrium bond lengths and harmonic vibrational frequencies of H_2_ and LiH are nearly identical with the experimental values. Both of the dissociation energies of H_2_ and LiH are slightly smaller than the corresponding experiment data. The small error of anharmonic constants for H_2_ and LiH are 0.8 cm^−1^ and 3.4 cm^−1^, respectively. Figure 1 shows the adiabatic and diabatic potential curves for a fixed internuclear distance of HH molecule (*r*_*HH*_ = 2.9 a_0_) as function of *R*_Li-HH_ for various values of *θ*, which is the angle between *R*_Li-HH_ and *r*_*HH*_. It is noted that the cross point shifts to the smaller *R*_Li-HH_ with the increase of *θ*. At large *θ*, the adiabatic potentials strongly avoid each other in the neighborhood of the crossing point; on the contrary, the diabatic potentials cross over with each other smoothly. Furthermore, in all cases, the adiabatic and diabatic energies become identical when the molecular geometries are far from the crossing point. Figure 2 shows the corresponding mixing angles which were used to constructed diabatic potential curves shown in Fig. 1. It can be seen from this figure that in the vicinity of the crossing point, the change rate of the mixing angle becomes steeper with the increase of *θ*. The mixing angle reaches an asymptotic value gradually with the increase of *R*_Li-HH_. Otherwise, according to Eqs (2) and (3), the cross point appears when the mixing angle *θ* equals to 45° and this conclusion is in agreements with the result of Fig. 1. Figure 3 shows the coupling potentials obtained from the corresponding mixing angle and the adiabatic potentials. The value of the coupling potential decreases more rapidly with the increase of *θ* and the coupling potential gradually decrease to zero, while the *R*_Li-HH_ increases to a large value. According to Eq. (4), it is can be concluded that the smaller value of the difference between 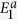 and 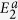 is, the smaller value of coupling potential is. This conclusion is consistent with the result obtained from Fig. 3. Figure 4 shows the adiabatic and diagonal diabatic potential energy curves for *θ* = 90° as a function of *R*_*LiH*_for various HH bond lengths *r*_*HH*_. It clearly shows that the cross point shifts to larger *R*_Li-HH_ with the increase of HH bond length. The three-dimensional diabatic PESs and the corresponding contour plots of the differences 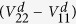 of the two diabatic states are presented in Fig. 5. From the upper panel of Fig. 5, it can be seen that for the top state, the reactant region was dominated by 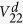 and the product region was dominated by 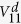, whereas the bottom state shows a reverse case. It can be seen that the transition of the title reaction occurs in the vicinity of interaction region. The lower panel of Fig. 5 shows the exact position of the crossing seam. The red lines in these contour plots represent the isopotential line 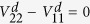, i.e., the conical intersection seam of the two surfaces. The Li(2p) + H_2_ → LiH(X^1^Σ^+^) + H reaction processes on the 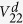 surface. The minimum energy paths of the title reaction as function of *R*_*HH*_ − *R*_*LiH*_ coordinate for various values of *θ* are shown in Fig. 6. From this plot it can be seen that there is a deep well (about 0.8 eV) along the reaction path. This well has a great impact on the reaction. The reactant zero point energy (ZPE) is 0.273 eV and the product ZPE is 0.086 eV, so that the endothermicity of the title reaction is 0.225 eV, taking into consideration the ZPEs of reactant and product.

### Dynamical calculations

**Figure 7:**
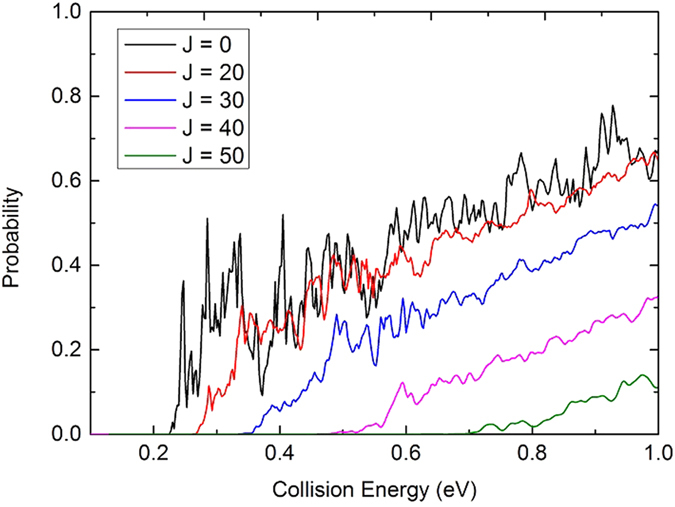
Total reaction probabilities of the Li(2p) + H_2_(X^1^Σ_g_^+^) → LiH(X^1^Σ^+^) + H reaction calculated by TDWP method at J = 0, 20, 30, 40 and 50.

**Figure 8:**
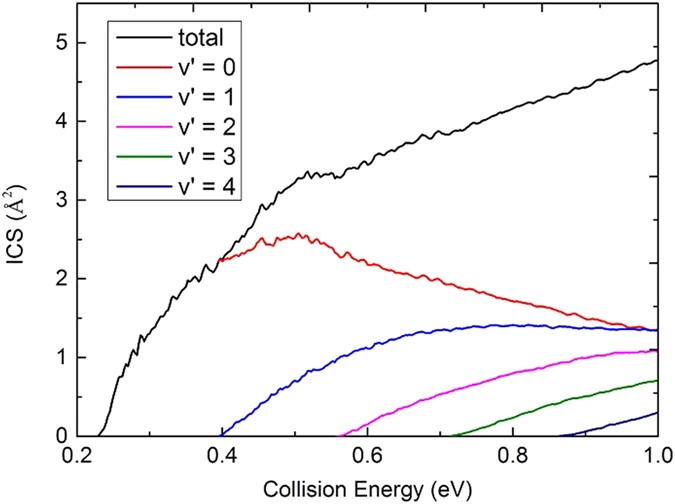
Total and vibrationally resolved ICSs of the Li(2p) + H_2_(X^1^Σ_g_^+^) → LiH(X^1^Σ^+^) + H reaction calculated by TDWP method.

**Figure 9:**
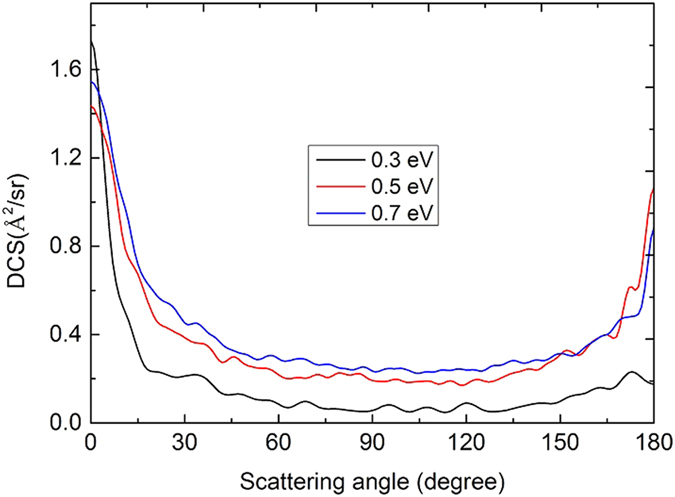
DCSs of the Li(2p) + H_2_(X^1^Σ_g_^+^) → LiH(X^1^Σ^+^) + H reaction calculated by TDWP method at three energies.

Figure 7 shows the initial state-specified (*v* = 0, *j* = 0) total reaction probabilities at five total angular momentum quantum number J values (0, 20, 30, 40, 50) as a function of the collision energy. For the reaction probabilities of J = 0, there is a threshold about 0.225 eV which corresponds to the endothermicity of the reaction. With the increase of the J value, the threshold increases due to the emergence of a centrifugal barrier. In the curves of the reaction probabilities, there are a mass of peaks, especially at low collision energies, which is a typical feature of quantum resonance. The feature probably corresponds to long-lived resonances associated with the well (about 0.8 eV relative to the Li(2p) + H_2_(X^1^Σ^+^_g_) asymptote) on the 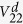. The amplitude of the oscillations decreases with the collision energy increasing, because of the decrease in lifetime of the intermediate complex. In TDWP calculation, the maximum total angular momentum quantum number is 65, which is large enough to calculate the integral cross sections (ICSs) and differential cross sections (DCSs) at the collision energies under 1.0 eV. Figure 8 shows the total and vibrationally resolved ICSs of Li(2p) + H_2_ (X^1^Σ^+^_g_) → LiH(X^1^Σ^+^) + H reaction calculated by the S-matrix method. There are a small amount of tiny oscillations on the ICSs curves, which is different from the intense and sharp peaks on the curves of probability. It is because the resonance structure is erased by summing over all the partial waves. For the total ICSs, the threshold is 0.225 eV which is consistent with the probability for J = 0. With the collision energy increasing, the total ICSs increases and the four vibrational excitation states of product appear in the order of v′ = 1 to v′ = 4. With the collision energy increasing, the ICS of ground vibrational state increases until reaching the maximum value at collision energy of 0.505 eV. When the collision energy increases from 0.505 eV, the ICSs for the vibrational excitation states still increases, but it decreases for the ground vibrational state. Therefore, the increase of the total ICSs comes from vibrational excitation states when the collision energy is higher than 0.505 eV. Figure 9 presents the DCSs of the Li(2p) + H_2_(X^1^Σ^+^_g_) → LiH(X^1^Σ^+^) + H reaction at three collision energies to study the angular distribution of the product LiH. As shown in the figure, the product molecule LiH tends to be forward scattering at low collision energy (0.3 eV). With the collision energy increasing, the peak at 180° appears, which implies that the trend of the backward scattering of LiH molecule becomes more and more obvious. However, the peak at 0° is still higher than that at 180°, thus the forward scattering is dominant in the title reaction.

## Discussion

A set of diabatic potential energy surfaces for the ground and first excited state of LiH_2_ system was constructed using a proper diabatization scheme and high-quality *ab initio* energy data. The diabatization scheme is based on transition dipole moments which could reflect the correct transition properties through the conical intersection. The *ab initio* calculations were performed by internally contracted multi-reference configuration interaction (MRCI)^28^,^29^ method. The augmented correlation-consistent polarization valence quadruple-ζ (AVQZ)^30^ atomic basis was employed for H atom and the Dunning-weighted correlation consistent polarized core-valence quadruple-ζ (WCVQZ)^30^ atomic basis was employed for Li atom. The NN methods are used to fit the diabatic potential energy surfaces and three high-accuracy potential surfaces were constructed with extremely small root mean square error (RMSE) (3.69 meV for 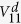, 5.34 meV for 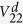 and 5.06 meV for 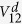). The endothermicity of Li(2p) + H_2_(X^1^Σ_g_^+^) → LiH(X^1^Σ^+^) + H reaction is 0.225 eV. There is a deep potential well about 0.8 eV exists in the reaction pathway. On the new PESs, the TDWP method was used to study the reaction of Li(2p) + H_2_ (X^1^Σ^+^_g_) → LiH(X^1^Σ^+^) + H. The reaction probabilities, ICSs and DCSs of the title reaction were calculated. The threshold is about 0.225 eV which corresponds to the endothermicity of the reaction. A mass of peaks are found on the curves of reaction probabilities especially at low collision energies, and this feature probably corresponds to the long-lived complex formed in the well. However, there is only a small amount of tiny oscillations on the total ICS curve, because the resonance is eased by summing over all the partial waves. The DCS results show the product LiH tends to be forward scattering at low collision energy (0.3 eV). With the collision energy increasing, the forward and backward scatterings both exist in the reaction. To our knowledge, there are no experimental results that can directly compare with theoretical results presented here. We are eagerly looking forward to a molecular beam experiment for the title reaction in the future, so that a comparison between theory and experiment can be fulfilled.

## Methods

### Potential Energy Surface

#### Diabatizaton scheme

The Li(2p) + H_2_ → LiH(X^1^Σ^+^) + H reaction is correlated with 1^2^A′ and 2^2^A′ of LiH_2_ molecule in *C*_*s*_ symmetry. For LiH_2_ system, the 1^2^A′ state corresponds to 1^2^A_1_ state in *C*_*2v*_ symmetry, while the 2^2^A′ state corresponds to 1^2^B_2_ state in *C*_*2v*_ symmetry. There are many methods of diabatization for the construction of the diabatic states^31^,^32^,^33^,^34^,^35^,^36^,^37^,^38^,^39^,^40^. In this work, the molecule property which could reflect the character of the transition between those coupled states is used to construct the diabatic PESs. The diabatization scheme used in this work is briefly described here and more detail presentation can be found in previous literatures^33^,^34^,^35^. Considering two coupling states, the diabatic wave functions 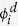 can be constructed from adiabatic wave functions 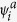 by the following unitary transformation


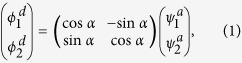


where *α* is the mixing angle. The diabatic energies 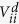 can be obtained in terms of adiabatic energies 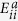 by


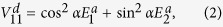



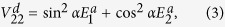



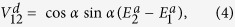



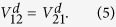


The 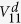 and 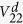 are the corresponding diabatic potential energies in the diabatic states, and the 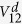 and 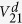 are the coupling potential energies between the two states. The mixing angle *α* can be obtained by molecular properties^35^,^36^,^37^,^39^,^40^. In our work, the transition dipole moments are used to obtain the mixing angle. According the Eq. (1), the matrix elements 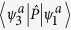 and 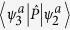 can be written as follows:


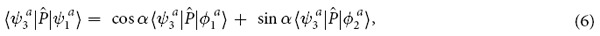



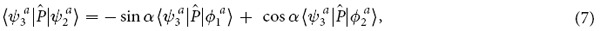


where 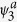 is a third state which not involved in the coupling. The operator 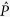 is taken to be dipole moment 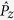 which is parallel to the ***z*** axis. An approximate treatment is that the 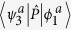 is set as zero and the 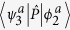 was set as one not just for the high symmetry geometries (*C*_*2v*_, *D*_*2h*_), but for all geometries. Then the mixing angle *α* can be derived from:


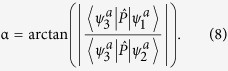


#### *Ab initio* calculations

The Jacobi coordinates are employed to generate the *ab initio* grid points. All of the three atoms are located in the ***x*****-*****z*** plane and the Li atom is fixed along the ***z*** axis. The center of mass of HH is set as the origin. The energy grid points are defined by 0.0 ≤ *R*_Li-HH_ */a*_0_ ≤ 30.0, 0.6 ≤ *r*_HH_*/a*_0_ ≤ 30.0 and 0.0 ≤ *θ/*deg ≤ 90 for Li-H_2_ reactant region, and 0.0 ≤ *R*_H-LiH_ */a*_0_ ≤ 30.0, 1.3 ≤ *r*_LiH_*/a*_0_ ≤ 30.0 and 0.0 ≤ *θ/*deg ≤ 180 for H-LiH product region, *R*, *r* and *θ* are the atom-diatom Jacobi coordinates. The coverage is sufficiently large for guaranteeing the quality of the asymptotic channels and the dynamic calculation. Otherwise, high-density grid points are distributed in connection region between strong interaction and asymptotic region for ensuring the accuracy of this region. In order to warrant the quality of the fitting, 15398 geometries are chosen to generate the *ab initio* energy points. Different step size of *R* and *r* are applied for generate the grid points. More specifically, the step size of *R* and *r* in interaction region, asymptotic region and connection region between the interaction and asymptotic region are about 0.2 Bohr, 2.0 Bohr and 0.5 Bohr, respectively. Moreover, the *ab initio* energy points above 20.0 eV relative to the energy of the Li-H-H dissociation limit are excluded from the fitting process. All of these *ab initio* energy points are calculated at the internally contracted MRCI level and using the complete active space self-consistent field (CASSCF)^41^,^42^ wave function as reference. Three valence electrons are included in eleven active orbitals and three states (1^2^A′, 2^2^A′ and 1^2^A″) of LiH_2_ are set equal weight in state-averaged CASSCF calculations. In both the CASSCF and MRCI calculations, the WCVQZ and the AVQZ basis sets are employed for the Li and H atoms, respectively. For the two states (1^2^A′, 2^2^A′) of LiH_2_, 30796 *ab initio* adiabatic energy points are obtained to construct the diabatic energies. The transition dipole moments mentioned above are also calculated. All *ab initio* calculations in this work are performed by MOLPRO^43^ package.

#### Fitting the diabatic PESs

The diabatic potential energies are obtained by the diabatization scheme mentioned above from the raw *ab initio* adiabatic energies. All of the diabatic potential energies are fitted by NN method. The back-propagation NN model is chosen in the fitting procedure. To ensure the quality of the fitting, there were two hidden layers in our NN method and 15 neurons were included in each hidden layer. There are 322 free parameters in the NN. The cross validation method was employed to detect and prevent over fitting of the training procedure. In this work, the *ab initio* energy points were separated into three sets, called training set, testing set and validation set. The training set was used for adapting the weights of the neural network and the validation set was used for early stopping of the training process. Moreover, the PESs have been checked by scanning the profiles for guaranteeing the non-physical behavior does not exist. A single neuron is a basic unit of the NN. Every neuron receives a set of input signals {*x*_*i*_} from the last layer and emits an output signal *y*_*i*_, which can be expressed as:


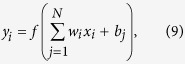


where *b*_*j*_ ( *j* = 1, 2, 3….*N*) are the bias and *w*_*i*_ are the connection weights between the two hidden layers. The transfer function *f*(*x*) is a critical factor for the quality of fitting the PES. The form of the transfer function in this work can be written as:


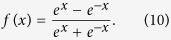


In order to include the permutation symmetry induced by the two H atoms, the permutation invariant polynomials^44^,^45^ are used in our fitting work. The final NN expansion can be presented as:


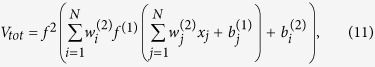


where the superscript represents the sequence number of different hidden layers. Finally, three high accuracy analytical PESs are constructed based on the diabatic energies and the RMSE of 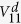, 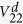 and 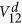 are 3.69, 5.34, 5.06 meV, respectively.

#### Reaction Dynamics

On the new diabatic PESs, TDWP calculation was performed for the Li(2p) + H_2_ (X^1^Σ^+^_g_) → LiH(X^1^Σ^+^) + H reaction. The TDWP method is a powerful tool to calculate initial state selected reactive collision and has been applied widely in many reactions^14^,^46^,^47^,^48^,^49^,^50^. A brief introduction of TDWP method which used here is given in this work and the detailed discussions can be found in relevant literature^48^,^49^. The TDWP method used in this work is based on the reactant coordinate based (RCB) approach and the method was developed by Sun *et al.*^49^. The basic theory RCB is to propagate an initial wave packet in the reactant Jacobi coordinates as in an initial state-selected total reaction probability calculation to obtain a scattering wave function for the product channels directly before the wave packet is absorbed in the product region. This method could treat the diabatic effect efficiently for studying non-adiabatic reaction, such as Cl + H_2_ reaction^51^. For a non-adiabatic reaction which take place over two coupled PESs, the Hamiltonian of the system is a 2 × 2 matrix. The off-diagonal elements of the matrix were employed to evaluate the transition probability between the diagonal elements. The Hamiltonian can be written as


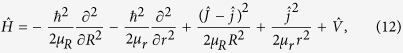


where *R* is the distance from the Li atom to the mass center of the H_2_ molecule and *r* is the bond length of the H_2_ molecule. *μ*_*R*_ and *μ*_*r*_ are the corresponding reduced masses. 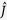 and 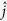 are the angular momentum operators of LiH_2_ system and reactant diatom molecule. 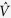 denotes the 2 × 2 matrix representation of the potential energies. The RCB method is used to extract the state-to-state S-matrix 
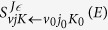
49. The state-to-state reaction probability is obtained by


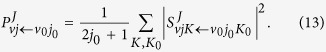


The state-to-state ICSs are calculated by


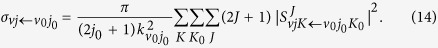


The DCSs are obtained by


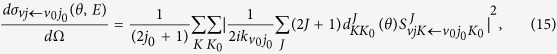


where *θ* is the scattering angle.

The initial rovibrational state of the reactant molecule H_2_ is set as *v*_0_ = 0, *j*_0_ = 0.The parameters used in the TDWP method are determined by numerous tests, and main parameters are listed in the Table 2.

## Additional Information

**How to cite this article**: He, D. *et al.* Global diabatic potential energy surfaces and quantum dynamical studies for the Li(2p) + H2(X^1^Σ^+^_g_) → LiH(X^1^Σ^+^) + H reaction. *Sci. Rep.* **6**, 25083; doi: 10.1038/srep25083 (2016).

## Figures and Tables

**Table 1:** Spectroscopic constants for LiH(X^1^Σ^+^) and H_2_(X^1^Σ_g_^+^).

		This work	Expt.^26^,^27^
H_2_(X^1^Σ^+^_g_)	*R*_*e*_ (Å)	0.7418	0.7414
	*D*_*e*_ (cm^−1^)	38186.2	38288.0
	*ω*_*e*_ (cm^−1^)	4402.7	4401.2
	*ω*_*e*_*x*_*e*_ (cm^−1^)	122.1	121.3
LiH(X^1^Σ^+^)	*R*_*e*_ (Å)	1.6018	1.5956
	*D*_*e*_ (cm^−1^)	20132.8	20287.7
	*ω*_*e*_ (cm^−1^)	1397.3	1405.1
	*ω*_*e*_*x*_*e*_ (cm^−1^)	26.6	23.2

**Table 2:** Numerical parameters used in the TDWP calculations. (Atomic units are used).

Li(2p) + H_2_(X^1^Σ^+^_g_) → LiH(X^1^Σ^+^) + H	
Grid/basis range and size	R ∈ [0.01, 15.0], N_*R*_ = 149
	r ∈ [0.01, 15.0], N_*r*_ = 149
	N_*j*_ = 69
Initial wave packet 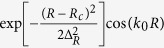	*R*_*c*_ = 10.0
	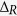 = 0.25
	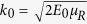 with *E*_0_ = 0.5 eV
Total propagation time	40000 iterations
Highest J value	65
